# Nocturnal Enuresis Associated With Sodium Valproate in an Adult Male With First Episode Psychosis: A Case Report

**DOI:** 10.1192/bjo.2026.11867

**Published:** 2026-06-30

**Authors:** Annesha Mukherjee

**Affiliations:** South West London and St George’s Mental Health NHS Trust, London, United Kingdom

## Abstract

**Aims:**

Sodium Valproate is widely used in the treatment of epilepsy and is also commonly prescribed for psychiatric disorders, particularly Bipolar Disorder. Enuresis associated with sodium valproate has been reported in paediatric populations with epilepsy, and in a case report involving a paediatric patient with Bipolar Disorder. However, data in the adult psychiatric population remains limited.

**Methods:**

This is a case report of a 20 year old gentleman diagnosed with Unspecified nonorganic psychosis. He had a four month psychiatric admission with first episode psychosis, which included 2 months in Psychiatric Intensive Care Unit (PICU). He was initially started on Olanzapine, which was titrated up to 20mg per day, however there was no response. Due to significant aggression, Sodium Valproate was introduced and increased up to 2500mg nocte. Due to lack of response with Olanzapine, antipsychotic medication was initially changed to Haloperidol during which he developed oculogyric crises which necessitated dose reduction. Due to persisting psychotic symptoms, it was changed to Zuclopenthixol. At discharge, he was on Zuclopenthixol depot 350mg every two weeks, Sodium Valproate 2500mg nocte and Procyclidine 5mg three times a day.

During follow up in the community by the Early Intervention Service, he was noted to have extrapyramidal side effects due to which the dose of Zuclopenthixol was reduced and Procyclidine was increased. He also reported nocturnal enuresis every night. There were no other urinary symptoms, and he had not had urinary incontinence or enuresis in the past. Initially, the dose of Sodium Valproate was split to 1000mg in the morning and 1500mg at night with a view to reduce nocturnal sedation, however the symptom persisted. The dose of Sodium Valproate was then reduced to 1000mg twice a day after which nocturnal enuresis resolved completely. During subsequent follow-ups, the dose of Sodium Valproate was reduced further, with a plan to gradually reduce and stop it in the long term. He remained free of psychotic symptoms during the period of follow up, without recurrence of enuresis.

**Results:**

The onset on nocturnal enuresis following initiation of Sodium Valproate, and resolution following dose reduction suggests a possible association between the two. This is in accordance with previous reports indicating enuresis as a dose related side effect of Sodium Valproate.

**Conclusion:**

Nocturnal enuresis is a potentially under recognised side effect of Sodium Valproate. Clinicians should screen for this adverse effect as it may significantly impact quality of life and treatment adherence.

